# Atypicalities in Perceptual Adaptation in Autism Do Not Extend to Perceptual Causality

**DOI:** 10.1371/journal.pone.0120439

**Published:** 2015-03-16

**Authors:** Themelis Karaminis, Marco Turi, Louise Neil, Nicholas A. Badcock, David Burr, Elizabeth Pellicano

**Affiliations:** 1 Centre for Research in Autism and Education (CRAE), Institute of Education, University of London, London, United Kingdom; 2 Department of Psychology, University of Florence, Florence, Italy; 3 Australian Research Council (ARC) Centre of Excellence in Cognition and Its Disorders, Department of Cognitive Science, Macquarie University, Sydney, Australia; 4 School of Psychology, University of Western Australia, Perth, Australia; Lyon Neuroscience Research Center, FRANCE

## Abstract

A recent study showed that adaptation to causal events (collisions) in adults caused subsequent events to be less likely perceived as causal. In this study, we examined if a similar negative adaptation effect for perceptual causality occurs in children, both typically developing and with autism. Previous studies have reported diminished adaptation for face identity, facial configuration and gaze direction in children with autism. To test whether diminished adaptive coding extends beyond high-level social stimuli (such as faces) and could be a general property of autistic perception, we developed a child-friendly paradigm for adaptation of perceptual causality. We compared the performance of 22 children with autism with 22 typically developing children, individually matched on age and ability (IQ scores). We found significant and equally robust adaptation aftereffects for perceptual causality in both groups. There were also no differences between the two groups in their attention, as revealed by reaction times and accuracy in a change-detection task. These findings suggest that adaptation to perceptual causality in autism is largely similar to typical development and, further, that diminished adaptive coding might not be a general characteristic of autism at low levels of the perceptual hierarchy, constraining existing theories of adaptation in autism.

## Introduction

Human perception will often assign causal qualities to perceptual events. For example, in a continuous sequence of events, object A moves towards a stationary object B, object A stops, and object B moves following the trajectory of object A. In this instance, we deduce that there was a collision, which *caused* B to move. This impression is less compelling if the stop position of object B overlaps with object A. In this case, we are more likely to infer that objects A and B move within different depths in the perspective of the visual scene and that the movement of object B is non-causal, i.e., unrelated to the movement of object A. This ability to form rapid causal judgements for perceptual events, referred to as *perceptual causality*, enables us to encode an inherently complex and dynamic physical environment as a coherent flux of related events [1, 2]. It is also an ability that is observed early in development—even 3-year-olds can use temporal cues to discriminate between causal and non-causal physical events [2], while habituation paradigms show that 9-month-old infants are sensitive to causality [3].

Many studies have suggested that perceptual causality relies upon low-level perceptual processes (‘early’ stages of vision), despite the fact that it involves inferences, which are typically associated with high-level cognition ([4, 5]; but see also [6], for alternative accounts). A recent study by Rolfs, Dambacher, and Cavanagh [1] provided evidence for the involvement of low-level processes in perceptual causality based on visual adaptation. Adaptation is a form of short-term neuronal plasticity, in which recently experienced stimuli affect the sensitivity of neurons, which in turn affects the perception of subsequent stimuli (e.g., [7]). Laboratory demonstrations of adaptation can be used to reveal neuronal populations specialised in the processing of particular dimensions of sensory input (e.g., colour).

Rolfs et al. [1] recently demonstrated a so-called negative adaptation aftereffect for perceptual causality in typical adults. After prolonged exposure (adaptation) to causal events, similar to the launches described in the example above, adults produced more judgments of perceptual events (test events) as non-causal. It was unlikely that the aftereffect was driven by other properties of the stimuli (e.g., size, timing of movements) because prolonged exposure to other similar stimuli did not create the impression of causality. The authors also considered conditions in which adaptation and test stimuli were presented in different combinations of spatial and retinal coordinates. An aftereffect appeared only when test events coincided with adaptation stimuli in retinal coordinates, that is, the effect was retinotopic. Retinotopy is suggestive of a reference frame shared by the retina and the visual cortex and is therefore a key characteristic of processes of the early stages of visual processing [8]. Rolfs et al.’s [1] retinotopic manifestation of the aftereffect provides evidence that low-level perceptual processes underpin the perception of causality in this task.

In the current study, we examined whether children with autism show adaptation to perceptual causality. Autism is a neurodevelopmental condition characterised by a range of social difficulties, as well as non-social symptoms, including repetitive behaviours and restricted activities and unusual reactions to sensory input. These latter sensory sensitivities, which include hypersensitivities to sensory input, as well as hyposensitivities and sensory seeking behaviours, which have only recently been included in the diagnostic criteria for autism (DSM-5; [9]), represent some of the most puzzling features of the condition [10].

There is renewed interest in these symptoms from researchers, prompted largely by the possibility that the sensory and other non-social symptoms of autism might be caused by fundamental differences in sensation and perception. We have suggested previously that atypicalities in adaptation, which is held to pose numerous functional advantages (e.g., [7]), might be one such difference [11, 12]. Adaptation helps to improve neuronal efficiency by dynamically tuning its responses to match the distribution of stimuli to make maximal use out of the limited working range of the system [13, 14, 15]. Any failure to continuously adapt to the current environment should also increase the transmission of redundant information, rendering one less able to distinguish irrelevant from relevant stimuli: which would have profound effects for how an individual might perceive and interpret incoming sensory information.

Research has shown much empirical support for this hypothesis—at least for high-level social stimuli. Children with autism have been found to show diminished adaptation in the coding of facial identity ([11]; though see [16], in adults with autism), facial configuration [17] and eye-gaze direction [18], while adults with autism have been found to present atypical adaptation to emotional categories (e.g., happy, sad; [19], but see also [16], for an account suggesting more general difficulties in the use of emotional labels). Adaptation to facial identity was also attenuated in relatives of children with autism compared with relatives of typical children, pointing towards the possibility of reduced adaptation as a potential endophenotype for autism [20].

These findings suggest that individuals with autism show diminished adaptation for high-level stimuli, at least those with social relevance. Since adaptation is ubiquitous in perceptual systems, these findings further raise the possibility that a reduced ability to adapt flexibly to incoming sensory input might be pervasive in autism.

Here we tested this possibility by examining adaptation to perceptual causality in children with autism. We chose perceptual causality for two reasons. First, and as discussed earlier, the retinotopic manifestation of the aftereffect in Rolfs et al. [1] provided definitive evidence that perceptual causality is indeed supported by mechanisms of low-level vision. Second, the fact that the low-level retinotopic brain areas in Rolfs et al. [1] paradigm detected and adapted to high-level dynamic features of the visual input, namely, causal interactions in dynamic scenes, was intriguing. Interestingly, Congiu, Schlottman, and Ray [21], have shown that school-age children with autism are just as able as their typically developing peers in recognizing perceptual causality (though see [22] for some difficulties in younger children with autism). An adaptation paradigm for perceptual causality would therefore allow us to focus on potential differences related to the adaptive coding of a process in low level-vision in school-age children with and without autism, rather than their ability to form causal judgements per se.

To this end, we developed a child-friendly paradigm for perceptual causality, similar to Rolfs et al. [1]. Our paradigm was based on simple animations with two identical squares, which could appear as causal (involving a collision causing a ‘launch’ movement) or non-causal (involving a ‘pass’ movement of one square next to the other square). During the adaptation phase, children received a period of prolonged exposure to causal events and were subsequently tested on their ability to perceive causality. If diminished adaptation in autism (i.e., [11, 18]) extends to low-level vision, then children with autism should show a negative aftereffect for perceptual causality (elevated rates of non-causal responses after prolonged exposure to causal events), albeit of a lesser degree than typical children of similar age and ability.

We were also careful to monitor attention during the task. Attention can impact upon the magnitude of the aftereffect [23, 24, 25]. In addition, as the aftereffect for perceptual causality in [1] was retinotopic (observed only when adapt and test stimuli were presented in the same retinotopic coordinates) it is essential that children maintain fixation. We controlled for attention and fixation by asking children to perform a secondary change-detection task (see also [26]), which required them to attend to a fixation point and report any colour changes during adaptation and test phases of the task. The change-detection task therefore allowed for the study of potential relations between the adaptive coding of perceptual causality and attention, at least as revealed by reaction times and accuracy in detecting colour changes.

## Method

### Participants

Participants’ demographics are shown in Table 1.

**Table 1:** Descriptive statistics for developmental variables for children with autism and typically developing children.

Measures	Children with autism	Typically developing children
**N**	22	22
**Gender** (n males: n females)	19: 3	11: 11
**Age (years)**		
Mean (SD)	11.16 (2.56)	11.08 (1.84)
Range	6.37–14.70	7.89–14.19
**Verbal IQ^a^**		
Mean (SD)	98.77 (12.59)	102.04 (9.43)
Range	74–122	89–131
**Performance IQ^a^**		
Mean (SD)	101.45 (15.03)	101.45 (11.76)
Range	75–128	88–129
**Full-Scale IQ^a^**		
Mean (SD)	99.95 (12.82)	101.45 (10.19)
Range	75–128	88–129
**SCQ score^b^**		
n	18	15
Mean (SD)	25.61 (8.31)	3.53 (2.39)
Range	11–44	0–9
**ADOS-G score^c^**		
n	18	0
Mean (SD)	9.33 (2.74)	n/a
Range	6–16	n/a

^a^Verbal, Performance and Full-Scale IQ were measured using the Wechsler Abbreviated Scales of Intelligence (WASI-II; [27])

^b^SCQ: Social Communication Questionnaire (score out of 40; [28])

^c^ADOS-G: Autism Diagnosis Observation Schedule-Generic (score out of 28; [29])

#### Children with autism

Twenty-two children with autism (19 boys) aged between 6 and 14 years (M = 11.16; SD = 2.56) were recruited via schools in London and community contacts. All children with autism had been previously diagnosed with autism (n = 15) or Asperger syndrome (n = 7) by independent clinicians. Children were administered the Autism Diagnostic Observation Schedule—Generic (ADOS-G; [29]; n = 18) and parents also completed the Lifetime version of the Social Communication Questionnaire (SCQ; [28]; n = 18), a screening test for autism (see Table 1 for scores). Children were included in data analysis if they had an independent clinical diagnosis of autism and scored above threshold for an autism spectrum disorder (ADOS-G cut-off score = 7; SCQ cut-off score = 15) in at least one of these two measures [30]. Thirteen children met criteria on both the ADOS-G and SCQ; four children met criteria on the ADOS-G only (including 3 whose parents did not complete the SCQ); and five children met criteria on the SCQ only (including 4 who did not complete the ADOS-G).

#### Typically developing comparison children

Twenty-two typically developing children (11 boys), recruited from local London schools, were individually matched with children with autism in terms of chronological age, t(42) = 0.10, p = 0.92, verbal IQ, t(42) = 0.68, p = 0.50, performance IQ, t(42) = 0.00, p = 1.00, and full-scale IQ, t(42) = 0.77, p = 0.45, as measured by the Wechsler Abbreviated Scales of Intelligence—2^nd^ edition (WASI-II; [27]). All children were therefore considered to be cognitively able (verbal IQ, performance IQ and full-scale IQ scores > = 70). There was a significant difference in terms of gender, **χ** ^2^(1, N = 44) = 6.70, p = 0.009, with more girls in the typical group than in the autism group.

Parents of typical children also completed the SCQ (n = 15). Their scores ranged between 0–9 (mean = 3.53, SD = 2.39), well below the cut-off point for autism [28].

#### Exclusions

Eight additional children with autism and 18 typical children (when forming a pool of 45 typically developing children, from which the comparison group was selected) were tested but excluded from the analysis due to poor psychometric functions (see below). The rate of exclusions of children with autism due to poor psychometric functions was comparable to such exclusions in typical children, **χ** ^2^(1, N = 94) = 0.08, p = 0.78. One further child with autism was excluded from the analysis due to poor performance in the change-detection task (0% accuracy in the post-adaptation phase, see below).

#### Ethics Statement

The study was conducted in accordance to the principles laid down in the Declaration of Helsinki. Parents of all children gave their informed written consent prior to their child’s participation in the project and children gave their verbal assent. The Institute of Education’s Faculty Research Ethics Committee approved all procedures (FPS456).

### Stimuli

Adapt and test stimuli were two grey squares, each 2.0 x 2.0 degrees of visual angle. The two squares were places symmetrically on the horizontal plane with a centre-to-centre separation of 8.0 degrees of visual angle. Vertically, they appeared in the top-half of the screen, 5.0 degrees of visual angle above a centrally located fixation point. Stimuli were presented in a 15.6-inch LCD monitor with 1366 x 768 pixel resolution at a refresh rate of 60 Hz and mean luminance of 60 cd/m^2^. All children viewed the stimuli binocularly from a distance of 57 cm from the screen. We wrote our experiments in Matlab, using the Psychophysics Toolbox extensions [31, 32, 33].

For the change-detection task (see below), the stimulus was a round fixation dot subtending 0.5 degrees of visual angle, which occasionally changed colour, from green to yellow.

### Procedure

We measured children’s judgements of perceptual causality with a child-friendly computer game that combined the primary causality-judgement task, and a secondary change-detection task, motivating children to attend to a fixation point at the centre of the screen. The general theme of the game was that children were members of a pirate crew, whose goal it was to recover the coins of a hidden treasure. The game consisted of three distinct phases (‘Levels’), a pre-adaptation phase, an adaptation phase, and a post-adaptation phase, administered in this order within a single session. Below, we provide an overview of the two tasks, including how these tasks were combined in the three phases of our paradigm.

#### Causality-judgment task

The perceptual causality task was based on simple animations (test events) in which two identical squares moved to the right side of the screen in a ‘relay race’ fashion: square A moving towards square B and stopping at a position that overlapped with square B; square B starting moving immediately after square A stopped, to the same direction and with the same speed as square A. These animations appeared either as causal (involving a ‘launch’ movement) or as non-causal (involving a ‘pass’ movement of the first square next to the other square), depending on the amount of overlap between the two squares in the joint position of the two movements. At the end of the animations, the child was asked to identify the final position of the first square, supposed to hide a coin. Note that the instructions for the perceptual causality task included no linguistic terms related to types of movements or causality (e.g., ‘square bumping onto/passing next to the other square’) and that the child’s judgements of perceptual causality were implicit (identifying a coin rather than labelling individual test events as ‘causal’ or ‘non-causal’).

During the adaptation phase, the child received a period of prolonged exposure to collision events (60 collisions, presented in pairs) and was subsequently retested on his/her ability to perceive causality (post-adaptation phase).

#### Change-detection task

In the change-detection task, the child was asked to respond to colour changes (from green to yellow) of the fixation point in centre-screen by pressing the spacebar. The fixation point returned to green after a response. In the pre-adaptation phase, colour changes of the fixation point occurred at the onset of each trial of the primary task, that is, each animation with the two squares. Responses were obligatory: the fixation point remained yellow (and the game did not move forward) until the child pressed the spacebar. This was to ensure that the test events of the perceptual causality task, which were presented subsequently (see detailed paradigm structure below), were shown after the child’s attention had been directed to the centre of the screen. In the adaptation phase, colour changes occurred probabilistically and during one third of the adaptation trials of the primary task, that is, during one third of the pairs of collision events. Unlike the pre-adaptation phase, the fixation point returned to its initial green colour even if the child did not press the spacebar (a missed colour change). This was to ensure an uninterrupted period of exposure to collision events. In the post-adaptation phase, each animation of the causality judgement task included one to three colour changes. Two colour changes occurred probabilistically, one third of the time, during top-up adaptation events (pairs of collisions) that preceded animations of the causality judgement task (see detailed structure below). Similar to the adaptation phase, the fixation point returned to green even if the children did not respond to colour change. A third colour change occurred before each post-adaptation test event. Similar to the pre-adaptation phase, the fixation point remained yellow until the child pressed the spacebar.

**Fig 1:**
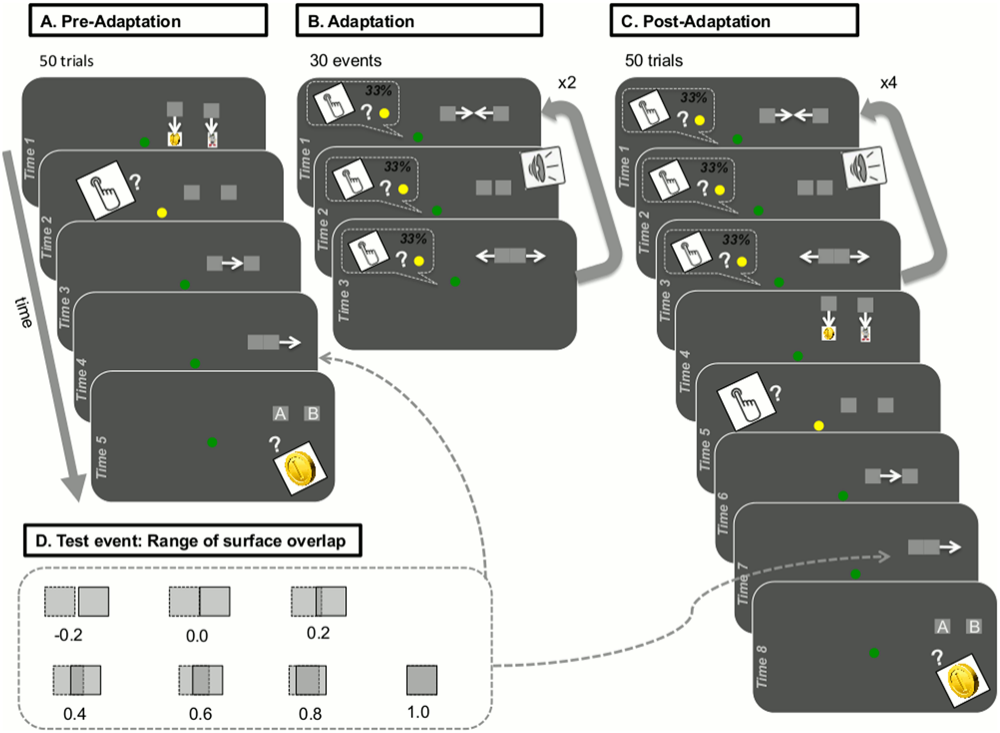
Task structure and stimuli presentation. A: Pre-Adaptation phase; B: Adaptation phase; C: Post-adaptation phase; D: Range of surface overlap in test events.

The detailed structure of the three phases of our paradigm, resulting from the combination of the perceptual causality judgement task and the change-detection task in our paradigm is shown in Fig. 1 and was as follows:

#### Pre-adaptation phase

In the pre-adaptation phase (‘Level 1’), the child was told s/he could gain coins in two ways, either by pressing the spacebar when the green dot in centre-screen turned yellow (change-detection task) or by identifying which of two squares had a coin in it (perceptual causality judgement task).

This phase included four demonstration trials and 50 test trials. Demonstration trials were used to ensure that the child understood the rules of the game and was able to respond correctly in easy conditions of the perceptual causality judgement task (values of surface overlap 0 and 1; see Fig. 1D). Unlike test trials, demonstration trials included feedback, as the true content of the two squares was revealed to the child after his/her response. Demonstration trials were repeated (after a random permutation) if the child produced incorrect judgements in at least two of those. This applied to eight children with autism and three typically developing children. No more than one repetition was required for nine of these children (all three typical children and six children with autism), while three children with autism received an additional repetition of practice trials.

At the beginning of each pre-adaptation trial (Fig. 1A), the child saw two squares moving to cover the images of a coin (left) and a recycle bin (right). Next, the green fixation point turned yellow, remaining so until the child responded to colour change by pressing the spacebar. The fixation point then returned to green, and the left square started moving towards the right square at a speed of 15 deg/sec. The left square stopped at a position that allowed for a targeted degree of surface overlap with the right square (‘test event’; Fig. 1D). Immediately after the left square stopped, the right square started moving in the same direction, with the same speed and covering the same distance as the left square. At the end of each pre-adaptation trial, both squares were at new positions (5.0 degrees of the visual angle above the centre, and 5.0 and 15.0 degrees of the visual angle to the right, for left and right square correspondingly). Two flashing labels, ‘A’ and ‘B’, also appeared on the left and the right square, respectively. The child pressed the A or the B key of the keyboard to indicate which square contained the coin.

The 50 test trials included random repetitions of seven conditions for the amount of surface overlap between the left squares when the movement of the left square stopped (-0.2, 0.0, 0.2, 0.4, 0.6, 0.8, and 1.0; see Fig. 1D). We expected the amount of surface overlap between the two squares to modulate the extent to which a given test event appeared as a ‘launch’ (causal movement of the second square) or a ‘pass’ (non-causal movement of first square). In trials where the surface overlap had values at the lower extreme of −0.2 or 0.0 (the left square not touching the right square when stopping or stopping immediately after touching the right square), the child should give ‘A’ or ‘square on the left’ responses in very high rates, that is, s/he should judge the test events mainly as causal (launches). In trials with substantial surface overlap with values at the upper extreme of 1.0 or 0.8 (the left square covering or almost covering the right square when stopping), the child should give high rates of ‘B’ or ‘square on the right’ responses, that is, judge test events mainly as non-causal (passes). The child’s judgements of test events should be less definite in trials using intermediate values of surface overlap (partial overlap between the two squares, when the left square stops).

#### Adaptation phase

In the adaptation phase (‘Level 2’; Fig. 1B), the child was instructed to press the spacebar when the green dot turned yellow. S/he was also advised to watch carefully as the game became progressively more difficult. The adaptation phase comprised 30 adaptation trials, where the two squares bounced back and forth towards each other at a speed of 15 deg/sec, and twice per trial, yielding 60 collisions in total. The fixation dot changed colour one third of the time (10 trials; see above). Adaptation trials had zero surface overlap between the two squares (the left square stopping immediately after touching the right square; see Fig. 1B), clearly appearing as causal movements [1]. To enhance this effect, collisions were also synchronised with a beep sound [34], 16ms in duration.

#### Post-adaptation phase

In the post-adaptation phase (‘Level 3’), the child was given exactly the same instructions on how to win treasure coins as in the pre-adaptation phase. The post-adaptation phase included 50 test trials (random repetitions of 7 values of surface overlap). Each post-adaptation trial (see Fig. 1C) began with top-up adaptation (4 collisions), which served to maintain adaptation [1]. Similar to the adaptation phase, the fixation point changed colour on one third of trials. The child responded by pressing the space bar as soon as s/he detected a colour change. After top-up adaptation, the child saw images of a coin and a recycling bin becoming covered by two new squares two new squares, and then the fixation point turned yellow, remaining so until they pressed the spacebar. As soon as the child responded to the colour change, the fixation point returned to green and the left square moved toward the right square, stopping next to it with a randomly selected degree of surface overlap. Next, the right square started moving towards the right hand side of the screen. At the end of the trial, labels ‘A’ and ‘B’ appeared on the two squares. The child indicated which of the two squares included a coin by making the appropriate keypress (‘A’ or ‘B’).

#### General procedure

Children were tested individually in a quiet room at the University, at school or at home. Testing on the experimental task lasted 15–20 min. The WASI-II and the ADOS-G were administered in later sessions.

## Results

### Causality-judgement task

**Fig 2:**
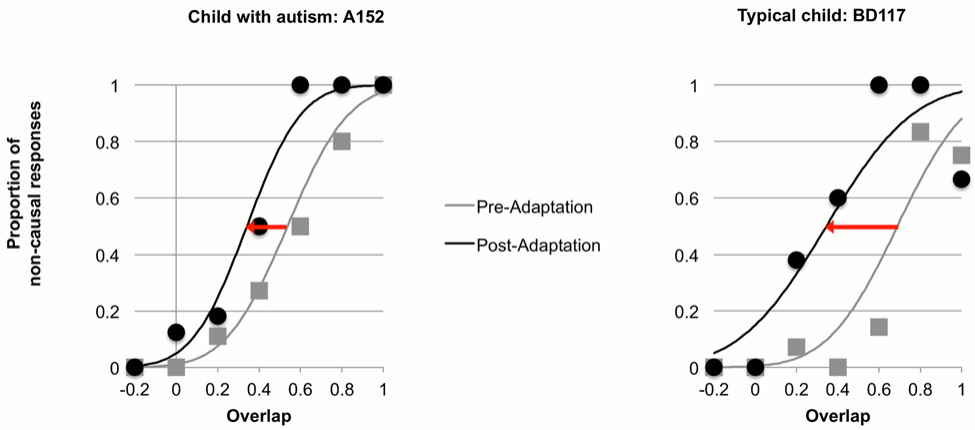
Example data and fitted psychometric functions in the pre- and post- adaptation testing conditions from the group of children with autism (left) and the group of comparison children (right). Proportion of non-causal (pass) responses is plotted as a function of surface overlap during the test event (Fig. 1D). The data were fitted with cumulative Gaussian error functions, whose means estimated PSE and standard deviations estimated precision. Red arrows indicate the shifting of psychometric functions during the post-adaptation phase.

**Fig 3:**
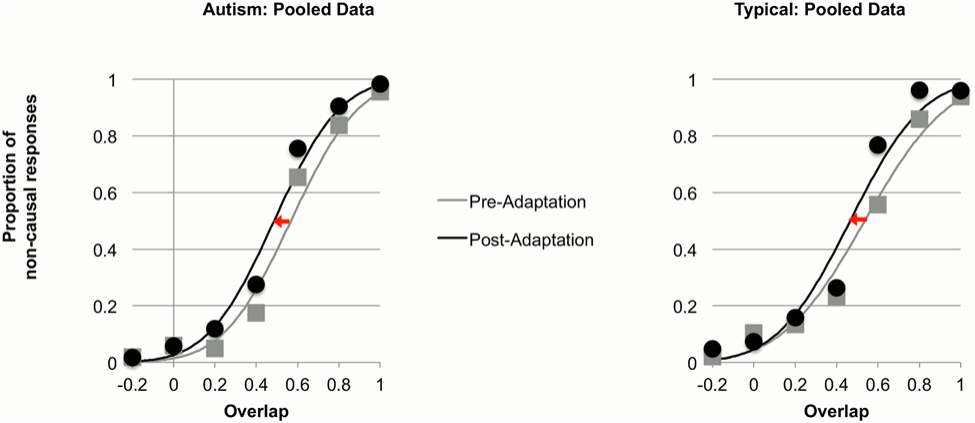
Pooled data across all participants within the group of children with autism (left) and the group of comparison children (right), and fitted psychometric functions in the pre- and post-adaptation testing conditions. Proportion of non-causal responses is plotted as a function of surface overlap during the test event (Fig. 1D). Red arrows indicate the shifting of psychometric functions during the post-adaptation phase.

Individual data from children with autism and typically developing comparison children were fit with cumulative Gaussian error functions (see example individual data in Fig. 2). Three observers, blind to any demographic details of the participants, examined all fitted curves initially to exclude children whose data was poorly-fit in either the pre- or post-adaptation phases (see Participants section above). Eight children with autism were excluded from the data analysis due to poorly-fitting cumulative Gaussian error functions, as well as 18 typical children. For children included in the analysis, their individual data were well fit by cumulative Gaussian functions (autism group: R^2^ = 0.94 ± 0.05; typical group: R^2^ = 0.94 ± 0.04). The fits for the pooled group data (Fig. 3) were excellent (both R^2^>.95).

As expected, adaptation to collision events caused elevated rates of non-causal responses (passes) in the post-adaptation condition. This pattern held for both groups and is evidenced in Figs. 2 (individual data) and 3 (pooled data) by the shifting of psychometric functions during post adaptation (red arrows). The position of the psychometric curves can be quantified by the point of subjective equality (PSE), defined as the mean of the cumulative Gaussian function (the point where 50% of the responses were non-causal). We defined the adaptation aftereffect as the difference between the PSEs in the pre-adaptation and the post-adaptation conditions: *PSEpre* −*PSEpost*.

**Fig 4:**
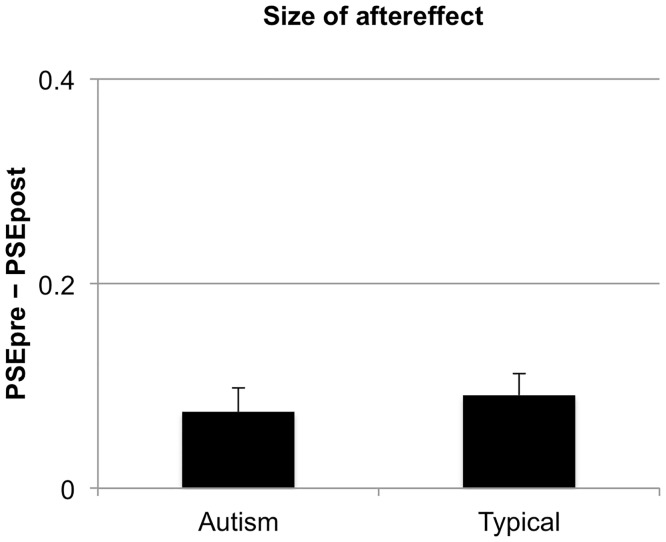
Size of the aftereffect for the groups of typically developing comparison children and children with ASD. The size of the aftereffect was defined as *PSEpre* −*PSEpost*, where *PSEpre* and *PSEpost* were the PSEs in the baseline and adapted conditions, correspondingly. Error bars correspond to ± 1 SEM.

Fig. 4 shows the magnitude of the aftereffect for children with autism (M = 0.07, SD = 0.11) and typically developing children (M = 0.09, SD = 0.10). Aftereffects were significant for both groups [autism: t(21) = 3.32, p = 0.003, typical: t(21) = 4.39, p < 0.001], in line with our prediction. Importantly, however, there were no significant group differences in the size of the aftereffect, t(42) = 0.52, p = 0.60, d = 0.19. Adaptation to perceptual causality in children with autism was found to be similar to such adaptation in typical children.

**Fig 5:**
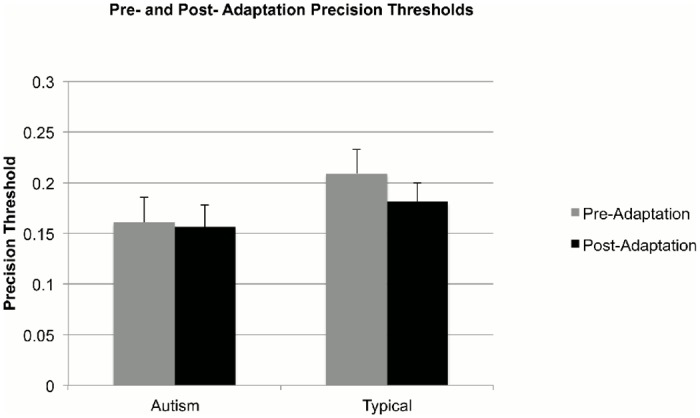
Mean precision for perception of causality (mean of standard deviations of the fitted psychometric curves) in the pre- and post-adaptation conditions for the group children with autism (left) and the group of typically developing comparison children (right). Error bars correspond to ± 1 SEM.

The psychometric functions provide not only an estimate of PSE, but also of precision thresholds, given by the standard deviation of the cumulative Gaussian functions. We therefore examined group differences in children’s precision in identifying causal and non-causal events. Precision thresholds in the pre- (autism: M = 0.16, SD = 0.11; typical: M = 0.20, SD = 0.11) and post- (autism: M = 0.16, SD = 0.10; typical: M = 0.18, SD = 0.08) adaptation conditions for the two groups of children are shown in Fig. 5. A mixed-design ANOVA, with condition (pre- and post- adaptation) as a repeated measures factor and group (autism and typical) as a between-participants factor, revealed no main effect of condition, F(1,42) = 0.73, p = 0.40, n_p_^2^ = 0.02, no significant main effect of group, F(1,42) = 2.27, p = 0.14, n_p_^2^ = 0.05, and no significant condition x group interaction, F(1,42) = 0.40, p = 0.53, n_p_^2^ = 0.01. This analysis suggested that children with autism were as precise as the comparison children in identifying causal and non-causal events in both the pre- and post-adaptation conditions.

Additional analyses with gender as a factor in the ANOVA showed no main effects of gender and no interactions involving gender either for the size of the aftereffect or on children’s precision thresholds (all ps > 0.26). We also examined potential developmental trends in children’s performance in the two tasks. When all data were combined, the magnitude of the aftereffect correlated positively with age, r(44) = 0.35, p = 0.02, suggesting the gradual emergence of the adaptation of perceptual causality between 6 and 14 years. When the data from each group were examined separately, these correlations did not reach significance (children with autism: r(22) = 0.38, p = 0.08; typical children: r(22) = 0.31, p = .15). There were no developmental improvements in precision in the perceptual causality task [pre-adaptation: r(44) = -0.22, p = 0.15; post-adaptation r(44) = -0.17, p = 0.26].

Finally, we examined the relationship between the size of the aftereffect and autistic children’s scores on measures of symptomatology. No significant correlations were found (ADOS-G: r(18) = 0.25, p = 0.31; SCQ: r(18) = –0.29, p = 0.25).

### Change-detection task

**Fig 6:**
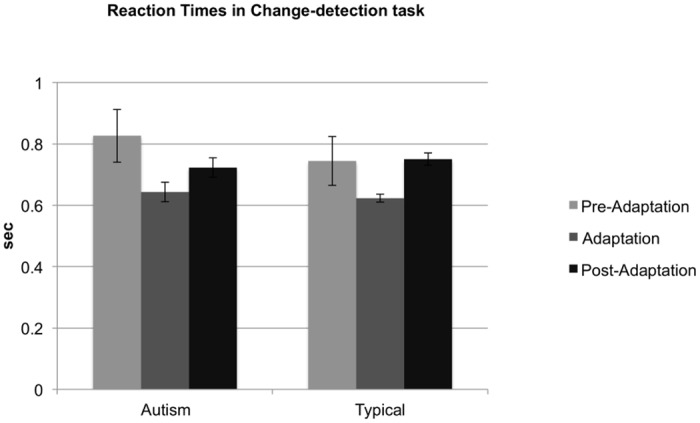
Mean reaction times in the change-detection task during the pre-adaptation, the adaptation and the post-adaptation phase for the group children with autism (left) and the group of typically developing comparison children (right). Error bars correspond to ± 1 SEM.

We analysed results from the change-detection task considering two measures, reaction times and accuracy. Average reaction times (secs) in the pre-adaptation phase (autism: M = 0.83, SD = 0.39; typical: M = 0.74, SD = 0.36), the adaptation phase (autism: M = 0.64, SD = 0.15; typical: M = 0.62, SD = 0.06) and post-adaptation (autism: M = 0.72, SD = 0.14; typical: M = 0.75, SD = 0.09) are shown in Fig. 6. We conducted a mixed-design ANOVA with condition (pre-adaptation, adaption, and post- adaptation) as a repeated measures factor and group (autism and typical) as the between-participants factor. There was a significant quadratic effect of condition [linear: F(1,42) = 0.80, p = 0.01, n_p_^2^ = 0.02; quadratic: F(1,42) = 18.88, p < 0.001, n_p_^2^ = 0. 95], which validated statistically that reaction times were faster in the adaptation phase. This effect is likely to be due to the adaptation phase being simpler, as it involved no judgements of perceptual causality. Importantly, the analysis of reaction times showed no significant effect of group, F(1,42) = 0.25, p = 0.62, n_p_^2^ = 0.01, and no significant interaction between group and condition [linear: F(1,42) = 1.01, p = 0.32, n_p_^2^ = 0.02; quadratic: F(1,42) = 0.02, p = 0.90, n_p_^2^ = 0.000]. Children with autism were therefore just as fast as typical children in their responses in the change-detection task.

**Fig 7:**
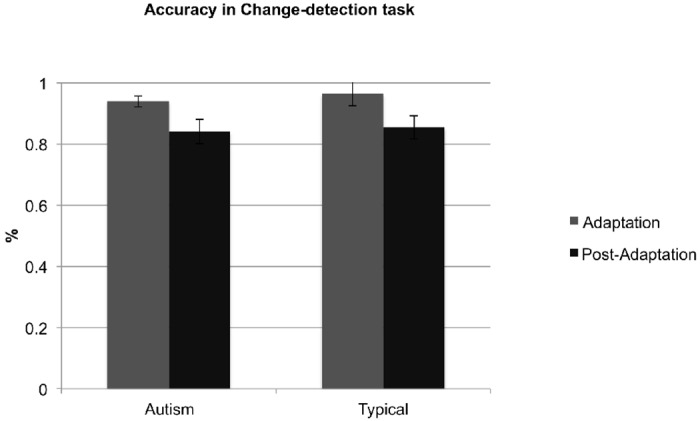
Mean accuracy in the change-detection task during the adaptation and the post-adaptation phase for the group children with autism (left) and the group of typically developing comparison children (right). Error bars correspond to ± 1 SEM.

We also evaluated children’s accuracy in detecting the colour changes that occurred in one third of the trials in the adaptation and post-adaptation phases (there were no probabilistic colour changes in the pre-adaptation phase). Accuracy rates for the two groups in the adaptation phase (autism: M = 93.94, SD = 8.20; typical: M = 96.46, SD = 8.66) and the post-adaptation phase (autism: M = 84.09, SD = 18.19; typical: M = 85.45, SD = 17.17) are shown in Fig. 7. These were significantly higher than chance [adaptation phase: autism, t(21) = 25.12, p < 0.001, typical, t(21) = 25.11, p < 0.001; post-adaptation phase: autism, t(21) = 8.79, p < 0.001, typical, t(21) = 9.69, p < 0.001], suggesting that children attended closely to the stimuli. A mixed-design ANOVA on accuracy rates with condition (adaptation and post- adaptation) as a repeated-measures factor and group (autism and typical) as a between-participants factor revealed a significant effect of condition, F(1,42) = 18.63, p < 0.001, n_p_^2^ = 0.31, suggesting that change-detection was easier during the adaptation phase. This result was also consistent with the faster reaction times in the adaptation phase. Again, there was no significant effect of group, F(1,42) = 0.32, p = 0.57, n_p_^2^ = 0.01, and no significant group x condition interaction, F(1,42) = 0.06, p = 0.81, n_p_^2^ = 0.001. Therefore, the accuracy rates of the two groups of children were indistinguishable. In summary, the results from the change-detection task suggested that children with autism were both as fast and as accurate in identifying the colour changes of the fixation point as typical children.

## Discussion

Previous studies on adaptation in autism have provided evidence for diminished adaptive coding of high-level (social) stimuli, including facial identity ([11]; see also [20], for similar findings in relatives of children with autism), facial configuration [17], and eye-gaze direction [18]. Since adaptation is ubiquitous in perceptual systems, we reasoned that diminished adaptive coding might be pervasive in autism and extend beyond faces. To test this possibility, we developed a child-friendly task to examine adaptation to perceptual causality in children with autism and typically developing children of similar age and ability. Our results indicate, contrary to our suggestion that children with autism adapted to perceptual causality to the same extent as typical children. The fact that children’s decision accuracy to the stimuli was similar across groups suggests that the results do not reflect inattention in our participants. These findings therefore suggest that diminished adaptation does not extend to low-level visual information.

We also found that the two groups were indistinguishable in precision in judging perceptual causality prior to adaptation. This finding is consistent with Congiu et al. [21], who found that children with autism recognized physical causality similarly to their typically developing peers. Ray and Schlottman [22], however, found poor recognition of causality in launch events in younger children with autism. These authors had attributed the difficulties children with autism had in recognizing causality in launch events to attentional limitations (e.g., [35]), which were critical in identifying short durations (specifically involved in launch events in their paradigm). Congiu et al. [21] argued that older children with autism might overcome these limitations with age. In our paradigm, we controlled for attention and found no differences between the two groups.

The size of the aftereffect in typical children was 0.09, compared to 0.27 in the original study by Rolfs et al. [1] with typical adults, potentially indicative of a weaker aftereffect in children. This difference could suggest that adaptation of perceptual causality is still maturing in the age range of our sample. However, it is also possible that the discrepancy in the results was due to methodological differences between the two studies including the number of trials, amount of adaptation and top-up adaptation, stimuli type—differences that were necessary to ensure that the current task was developmentally appropriate and engaging for children.

Our findings are also inconsistent with many prominent theories of autistic perception, such as the weak central coherence theory, which suggests reduced global processing in autism [36], and the enhanced perceptual functioning account [37], which posits that a local-processing bias leads to strengths in the processing of simple stimuli and to weaknesses in the processing of more complex stimuli. These two theories are similar in assuming difficulties in the integration of local sensory signals, which compromise the formation of global percepts in autism. In our task, judging perceptual causality in the animations required the efficient encoding of spatiotemporal contingencies in visual input presented in different areas of the screen. Our paradigm therefore involved complex stimuli and relied upon global processing. The finding that children with autism were indistinguishable from typically developing children in terms of their precision of perceptual causality judgments challenges accounts assuming difficulties in the integration of local sensory input in autism—at least at low perceptual levels.

Our study clearly shows that adaptation to perceptual causality is not attenuated in autism. This finding raises questions about the pervasiveness of diminished adaptation in autism. There are three potential possibilities.

One possibility is that earlier findings for diminished adaptation of faces do not generalize beyond facial stimuli. If this is the case, diminished adaptation, and possibly adaptive coding, might reflect face-reading difficulties that underlie many of the social difficulties in autism, rather than a general problem with adaptation. Many studies have shown a link between adaptation to face stimuli and face-processing abilities. For example, Dennett et al. [38], reported significant correlations between face aftereffects and face recognition abilities in typical adults. Also, Pellicano et al. [18] showed that being less accurate in categorizing subtle deviations of eye-gaze direction was linked to diminished adaptation to eye-gaze in children with autism. In Pellicano et al. [11], however, children with autism were less adaptable to face-identity, but as precise as typically developing children of similar age and ability in identifying faces. Possible reasons for these discrepancies could be the simplicity of the discrimination required (between only two identities) and the extensive training of the participants in Pellicano et al. [11].

A second possibility is that diminished adaptation is not limited to faces, but extends to high-level *social* stimuli. This could be tested using adaptation paradigms that distinguish between face-processing and social difficulties in autism, for instance, paradigms addressing the perception of biological motion (typically using point-light-display representations of human actions, based on key anatomical points, e.g., [39]). The efficient processing of biological motion is important for a wide range of social abilities, such as inferring other people’s emotions, mood, and intentions (e.g., [40]). Neuroimaging studies have suggested that biological motion is supported by high-level neuronal mechanisms within the superior temporal gyrus (STS), a brain area that is also involved in the processing of faces [41], as well as the extrastriate and fusiform body areas (EBA and FBA; [42]). The perception of biological motion in typical development is also subject to adaptation, for example, to the gender of a walking figure [43, 44]. Individuals with autism present reduced sensitivity to biological motion and differences in the brain activation patterns following the presentation of relevant biological stimuli [45, 46, 47]. It would be of great interest to study whether individuals with autism also present diminished adaptation to biological motion.

A third possibility is that diminished adaptation of high-level processes is indeed a general property of autism, including social *and* non-social processes, but not the specific adaptation of “causation”. Indeed there is some suggestion [48] that the particular paradigm of Rolfs et al. [1] does not examine adaptation of causality per se, but adaptation of a more specific attribute, such as perceived malleability. Thus it is possible that high-level processes do, in general, show reduced adaptation in autism.

This possibility can be tested using paradigms that tap non-social processes of high-level vision, for example, numerosity. Numerosity is supported by a composite of different processes, including basic perceptual and high-level brain networks (e.g., temporal and parietal regions; [49, 50]). Findings that numerosity is subject to adaptation [51] are suggestive of a primary visual process, which is independent of mechanisms related to visual features, such as texture perception [52]. The ability to estimate numerosity in visual scenes is less likely to contribute to social abilities, and indeed anecdotal reports have suggested superior number sense abilities in autism (though see [50], for poorer abilities for large numerosity estimation in adults with autism). Preliminary results from our laboratory [53] suggest that adaptation to number is reduced with autism.

Our results highlight the need for a more nuanced account for adaptation in autism, explaining in particular the potentially uneven adaptation profile in autism. Such an account will need to be based on evidence from the contrastive study of adaptation across different stimuli dimensions, including low-level vs. high-level vision, social vs. non-social stimuli, face-related vs. non-face-related social stimuli, and combinations of these. Addressing this issue will be highly informative for our understanding of autistic perception, as well as the mechanisms that underlie visual adaptation more broadly.
